# *Lactobacillus plantarum* C88 protects against aflatoxin B_1_-induced liver injury in mice via inhibition of NF-κB–mediated inflammatory responses and excessive apoptosis

**DOI:** 10.1186/s12866-019-1525-4

**Published:** 2019-07-29

**Authors:** Li Huang, Zijian Zhao, Cuicui Duan, Chao Wang, Yujuan Zhao, Ge Yang, Lei Gao, Chunhua Niu, Jingbo Xu, Shengyu Li

**Affiliations:** 1 School of Environment, Northeast Normal University, No. 2555 Jing-Yue Street, Changchun, Jilin Province 130117 People’s Republic of China; 2 Institute of Agro-food Technology, Jilin Academy of Agricultural Sciences, No. 1363 Sheng-Tai Street, Changchun, Jilin Province 130033 People’s Republic of China

**Keywords:** Lactic acid Bacteria, *Lactobacillus plantarum*, Aflatoxin B_1_, Liver injury, NF-κB signal pathways, Anti-inflammatory, Apoptosis

## Abstract

**Background:**

Probiotics play an important role in the human and animal defense against liver damage. However, the protective mechanism of *Lactobacillus plantarum* C88 on chronic liver injury induced by mycotoxin remains unclear.

**Results:**

In this study, the addition of *L. plantarum* C88 obviously ameliorated the increased contents of alanine aminotransferase (ALT), aspartate aminotransferase (AST), alkaline phosphatase (ALP), total cholesterol and triglyceride, the diminish contents of total protein and albumin in serum of mice challenged with AFB_1_. Simultaneously, *L. plantarum* C88 attenuated the inflammatory response via significantly reducing the levels of pro-inflammatory factors, including interleukin-1β (IL-1β), IL-6, IL-8, interferon-γ (IFN-γ) and tumor necrosis factor-α (TNF-α) in serum. Furthermore, *L. plantarum* C88 remarkably down-regulated the nuclear factor kappa B (NF-κB) signaling pathways by weakening the expression of toll-like receptor 2 (TLR2) and TLR4, and inhibited NF-κB nuclear translocation through enhancing the expression of NF-κB inhibitor (IκB). Neutralization experiments confirmed that *L. plantarum* C88 decreased the levels of some pro-inflammatory factors due to the suppression of the NF-κB signaling pathways. Besides, *L. plantarum* C88 decreased the levels of Bax and Caspase-3, elevated the level of Bcl-2, and reduced mRNA expressions of Fatty acid synthetase receptor (Fas), FAS-associated death domain (FADD), TNF receptor associated death domain (TRADD) and Caspase-8 in the liver.

**Conclusions:**

Probiotic *L. plantarum* C88 prevented AFB_1_-induced secretion of pro-inflammatory cytokines by modulating TLR2/NF-κB and TLR4/NF-κB pathways. The molecular mechanisms of *L. plantarum* C88 in ameliorating AFB_1_-induced excessive apoptosis included regulating the mitochondrial pathway and cell death receptor pathways.

**Electronic supplementary material:**

The online version of this article (10.1186/s12866-019-1525-4) contains supplementary material, which is available to authorized users.

## Background

As an important detoxification organ, the liver is continuously exposed to certain adverse factors such as alcohol, fat, pathogens and cellular metabolites, which can cause liver injury, hepatitis and liver degradation. Long-term liver injury leads to liver fibrosis, cirrhosis and hepatocellular carcinoma (HCC) [1]. Clinically, the main causes of liver injury are viruses, alcohol, drugs, and chemicals. Liver injury can also be induced by toxins, such as AFB_1_. AFB_1_ is a metabolite of *Aspergillus flavus* and *A. parasiticus*, which is classified under Group I carcinogenic agents by International Agency for Research on Cancer (IARC) [2], and is a potent hepatotoxic and hepatic carcinogenic mycotoxin in humans. The mechanisms of AFB_1_-induced liver injury are complex, and previous studies have shown that inflammatory response is a critical step in the process. Mehrzad et al. [3] found that AFB_1_ activates TLR2 and TLR4, which trigger the NF-κB signaling pathway that leads to the synthesis and secretion of TNF-α, IL-1β, IL-6 and other pro-inflammatory cytokines in murine pure primary astrocytes. Therefore, it is necessary to find safe and effective natural anti-inflammatory agents for preventing or alleviating AFB_1_-induced hepatic injury.

Probiotics are used as a dietary supplement for their outstanding health benefits including immunoregulation, balancing lipid metabolism, and regulation of gene expression in diseases such as inflammatory bowel disease, alcoholic and non-alcoholic liver diseases. *L. plantarum* is effective in preventing chronic and acute alcohol-induced liver injury by improving intestinal barrier function [4], restoring gut microbiota [4], regulating pro-inflammatory molecules [5], reducing oxidative stress [6], and disrupting endotoxemia [7]. Meanwhile, *L. plantarum* mitigates non-alcoholic fatty liver disease by improving liver function, inducing adipose leptin, enhancing antioxidant capacity and decreasing lipid peroxidation [8, 9]. *L. plantarum* could also resist liver damage induced by endotoxins, *D*-galactosamine, lipopolysaccharides and cadmium by a similar pathway [10–13].

*L. plantarum* C88 is classified as a probiotic since it colonizes the gastrointestinal tract [14], resists bile salt or aciduricity, possesses antioxidant capacity [15], prevents chronic and acute liver injury induced by alcohol [16, 17], and modulates the metabolism of AFB_1_ in mice [18]. However, the effects of *L. plantarum* on AFB_1_-induced hepatic inflammation and excessive apoptosis are rarely reported. This study aimed to further reveal the molecular mechanisms of *L. plantarum* C88 on hepatic injury using the AFB_1_ challenge mouse model.

## Results

### Inhibition of AFB_1_-induced liver injury

As depicted in Table 1, Additional file 1 the levels of serum ALT, AST and ALP of the AFB_1_ model group were significantly increased (*P* < 0.05), along with similar increase in the total cholesterol and triglyceride levels (*P* < 0.05). As compared to the control group, the total protein and albumin levels were increased in the viable C88 and heat-killed C88 groups, while the total cholesterol and triglyceride levels were decreased, although without significant differences. The therapy group mice, treated with a combination of AFB_1_ and *L. plantarum* C88, successfully recovered the serum biochemical parameters to the levels of the control group. This recovery was significantly pronounced in the AFB_1_ + Viable C88 group.

**Table 1:** Effect of *L. plantarum* C88 on serum biochemical parameters

Group	ALT (U/L)	AST (U/L)	ALP (U/L)	Total cholesterol (mmol/mL)	Triglycerides (mmol/mL)	Total protein (g/L)	Albumin (g/L)
Control	40.25 ± 2.98 ^c^	64.79 ± 6.79 ^c^	106.54 ± 7.52 ^c^	135.89 ± 10.59	87.69 ± 7.92 ^c^	79.68 ± 6.40 ^a^	50.44 ± 5.63 ^b^
AFB_1_	89.78 ± 4.13 ^a^	120.61 ± 9.31 ^a^	194.39 ± 12.64 ^a^	283.13 ± 17.22 ^a^	191.83 ± 15.02 ^a^	45.77 ± 4.03 ^c^	38.85 ± 4.94 ^c^
Viable C88	39.89 ± 2.67 ^c^	56.88 ± 7.95 ^c^	86.36 ± 7.95 ^c^	110.92 ± 10.23 ^c^	39.50 ± 4.63 ^d^	88.85 ± 7.41 ^a^	65.83 ± 6.93 ^a^
Heated-killed C88	40.14 ± 2.92 ^c^	57.40 ± 6.83 ^c^	92.40 ± 6.71 ^c^	118.23 ± 12.49 ^c^	50.82 ± 7.21 ^d^	80.71 ± 4.46 ^a^	53.67 ± 4.62 ^b^
AFB_1_ + Viable C88	69.14 ± 3.34 ^b^	82.92 ± 9.14 ^b^	154.02 ± 14.14 ^b^	154.28 ± 13.51 ^b^	111.64 ± 10.64 ^b^	65.16 ± 5.66 ^b^	45.77 ± 5.62 ^b^
AFB_1_ + Heated-killed C88	76.72 ± 3.94 ^b^	101.53 ± 8.25 ^b^	181.80 ± 11.76 ^a^	179.91 ± 10.24 ^b^	118.27 ± 8.93 ^b^	47.32 ± 4.68 ^c^	42.02 ± 3.29 ^c^

The results are expressed as mean ± S.D.; each data point is the average of 3 repeated measurements from 10 independently replicated experiments (*n* = 10)

The different letters in the same rows mean significant difference (*P* < 0.05), the same letters in the same rows mean insignificant difference (*P* > 0.05)

### Reduction of inflammatory response

The effects of different treatments on pro-inflammatory cytokines in serum are presented in Table 2, Additional file 2. In the AFB_1_ group, the levels of IL-1β, IL-6, IL-8, IFN-γ and TNF-α in serum were increased as compared to the control group (*P* < 0.05). However, these increases were prevented in the AFB_1_ + Viable C88 group or AFB_1_ + Heat-killed C88 group, suggesting that *L. plantarum* C88 supplementation could restrain the inflammatory processes in serum caused by AFB_1_. In addition, the levels of IL-10 showed no significant differences among the groups (*P* > 0.05).

**Table 2:** Effect of *L. plantarum* C88 on the levels of IL-1β, IL-6, IL-8, IL-10, IFN-γ, and TNF-α in serum

Group	IL-1β (pg/ml)	IL-6 (pg/ml)	IL-8 (pg/ml)	IL-10 (ng/ml)	IFN-γ (pg/ml)	TNF-α (pg/ml)
Control	43.45 ± 3.90 ^c^	67.24 ± 8.35 ^c^	110.26 ± 15.02 ^c^	161.79 ± 9.93 ^a^	163.24 ± 17.90 ^b^	208.85 ± 33.03 ^c^
AFB_1_	99.23 ± 5.52 ^a^	123.56 ± 12.73 ^a^	143.40 ± 11.80 ^a^	153.28 ± 10.90 ^a^	252.55 ± 19.65 ^a^	313.53 ± 44.17 ^a^
Viable C88	41.52 ± 4.34 ^c^	74.83 ± 8.49 ^c^	91.77 ± 15.58 ^d^	168.72 ± 8.46 ^a^	169.68 ± 17.81 ^b^	204.39 ± 31.40 ^c^
Heated-killed C88	42.49 ± 4.21 ^c^	72.35 ± 9.83 ^c^	98.41 ± 6.83 ^d^	166.97 ± 10.49 ^a^	166.88 ± 16.29 ^b^	208.37 ± 29.18 ^c^
AFB_1_ + Viable C88	68.82 ± 3.72 ^b^	109.24 ± 13.51 ^b^	128.11 ± 13.21 ^b^	155.80 ± 7.28 ^a^	236.83 ± 20.53 ^a^	269.04 ± 24.65 ^b^
AFB_1_ + Heated-killed C88	79.25 ± 4.56 ^b^	113.52 ± 11.69 ^b^	130.29 ± 11.77 ^b^	153.29 ± 9.84 ^a^	241.01 ± 21.88 ^a^	278.23 ± 33.42 ^b^

The results are expressed as mean ± S.D.; each data point is the average of 3 repeated measurements from 10 independently replicated experiments (*n* = 10)

The different letters in the same rows mean significant difference (*P* < 0.05), the same letters in the same rows mean insignificant difference (*P* > 0.05)

### Decreased apoptosis of liver

**Fig. 1:**
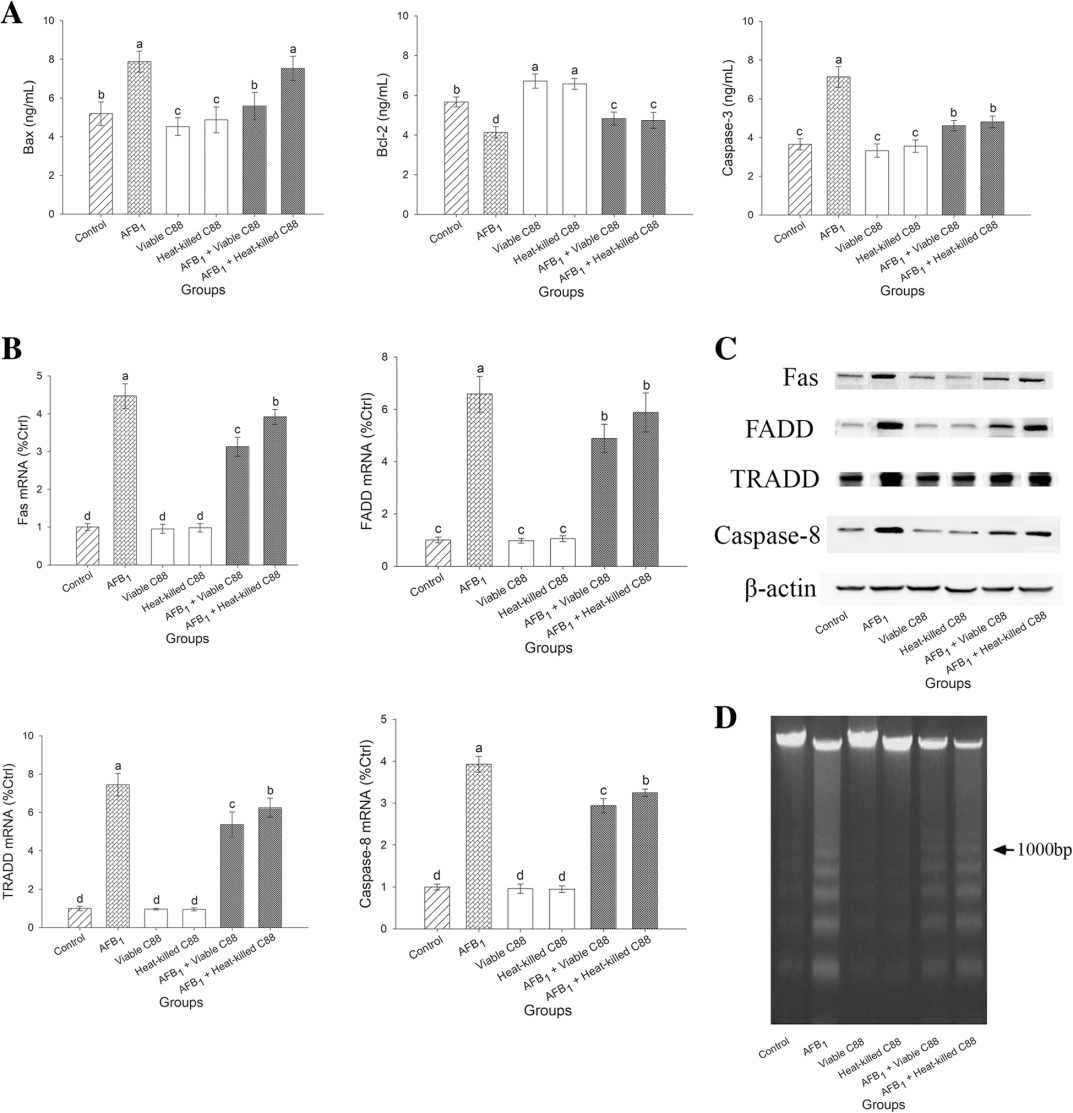
Effect of *L. plantarum* C88 on decreased apoptosis of liver. **a** The levels of Bax, Bcl-2 and Caspase-3 in liver were measured. **b** FAS, FADD, TRADD and Caspase-8 gene expression was measured by RT-PCR. **c** Expression of FAS, FADD, TRADD, Caspase-8 and β-actin was detected by Western blot analysis. β-actin was used as a housekeeping control. **d** DNA was extracted and analyzed by agarose gel electrophoresis to analyze the DNA fragmentation. The different letters in the same rows mean significant difference (*P* < 0.05), the same letters in the same rows mean insignificant difference (*P* > 0.05).

In comparison with the AFB_1_ model group, the levels of Bax and Caspase-3 were significantly reduced in the AFB_1_ + Viable C88 group or AFB_1_ + Heat-killed C88 group (*P* < 0.05), although they did not recover to the levels in the control group (Fig. 1a, Additional file 3). The addition of *L. plantarum* C88 to the AFB_1_ diet significantly restored the reduction of Bcl-2 in the liver (*P* < 0.05).

The mRNA and protein expression levels of relative genes in liver involved in death receptor pathway, including FAS, FADD, TRADD and Caspase-8, are presented in Fig. 1b and c. AFB_1_ significantly up-regulated the mRNA expression levels of FAS, FADD, TRADD and Caspase-8 in the liver (*P* < 0.05). However, addition of *L. plantarum* C88 to AFB_1_ diet significantly reversed the increase in mRNA expression of above genes caused by AFB_1_ treatment (*P* < 0.05). The mRNA expression of FADD was down-regulated, although not significantly, in the AFB_1_ + Heat-killed C88 group (*P* > 0.05). Western blotting also revealed that AFB1 exposure significantly up-regulated the death receptor pathway as a consequence of 21 days of AFB_1_ exposure, while *L. plantarum* C88 pretreatment inhibited the expression of FAS, FADD, TRADD and Caspase-8 (Fig. 1c).

Genomic DNA fragmentation was assayed to confirm the occurrence of hepatocyte apoptosis. An inconspicuous DNA fragmentation occurred in the control group, while no DNA fragmentation was found in viable and heat-killed *L. plantarum* C88 groups (Fig. 1d). Maximum DNA fragmentation was observed in AFB_1_ group, while co-treated with AFB_1_ and *L. plantarum* C88 was able to inhibit the charac-teristic apoptotic DNA fragmentation.

### Regulation of NF-κB signaling pathway

The NF-κB signaling pathways are involved in the inflammatory response and cell injury. The mRNA expression of NF-κB was significantly upregulated in the liver of mice fed with AFB_1_ diet (*P* < 0.05). The increased mRNA expression of NF-κB was highly inhibited in the therapy group of AFB_1_ + *L. plantarum* C88. Since NF-κB is activated after its nuclear translocation, Western blot analysis was performed using antibodies to the p65 subunit of NF-κB to assess the distribution of NF-κB in liver tissue. A significant increase in NF-κB protein expression was observed in the nucleus of the mice fed with AFB_1_, in conjunction with a decrease in IκB level, indicating a nuclear translocation from the cytoplasm. The addition of *L. plantarum* C88 to the AFB_1_ diet prompted a considerable down-regulation in protein expression of NF-κB.

**Fig. 2:**
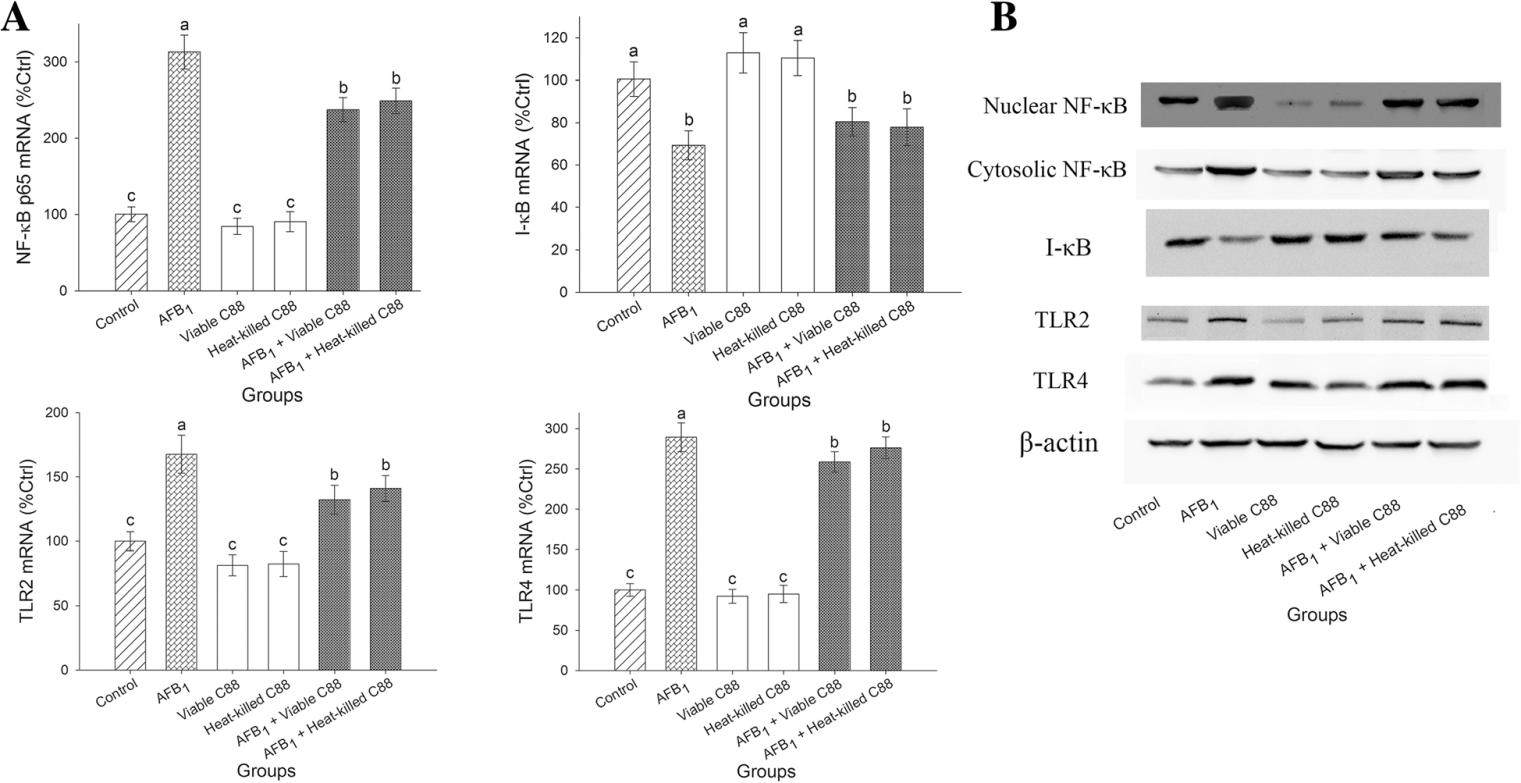
Effect of *L. plantarum* C88 on NF-κB signaling in liver of mice. **a** NF-κB, I-κB, TLR2, and TLR4 gene expression was measured by RT-PCR. **b** Expression of Nuclear NF-κB and Cytoplasmic NF-κB, I-κB, TLR2,TLR4 and β-actin was detected by Western blot analysis. β-actin was used as a housekeeping control. The results are expressed as mean ± S.D.; each data point is the average of 3 repeated measurements from 10 independently replicated experiments (*n* = 10). The different letters in the same rows mean significant difference (*P* < 0.05), the same letters in the same rows mean insignificant difference (*P* > 0.05)

TLR2 and TLR4 are important signaling receptors for activating NF-κB signaling pathway. In mice exposed to the AFB_1_ diet, the mRNA expression levels of TLR2 and TLR4 were up-regulated in the liver, and were nearly 1.67-fold and 2.89-fold those of the control group, respectively. However, in the therapy groups, the mRNA expression levels of TLR2 and TLR4 were significantly decreased (*P* < 0.05), and the decrease in protein expression levels were also confirmed by Western blot (Fig. 2).

### NF-κB pathway stimulates the synthesis of pro-inflammatory cytokines

**Fig. 3:**
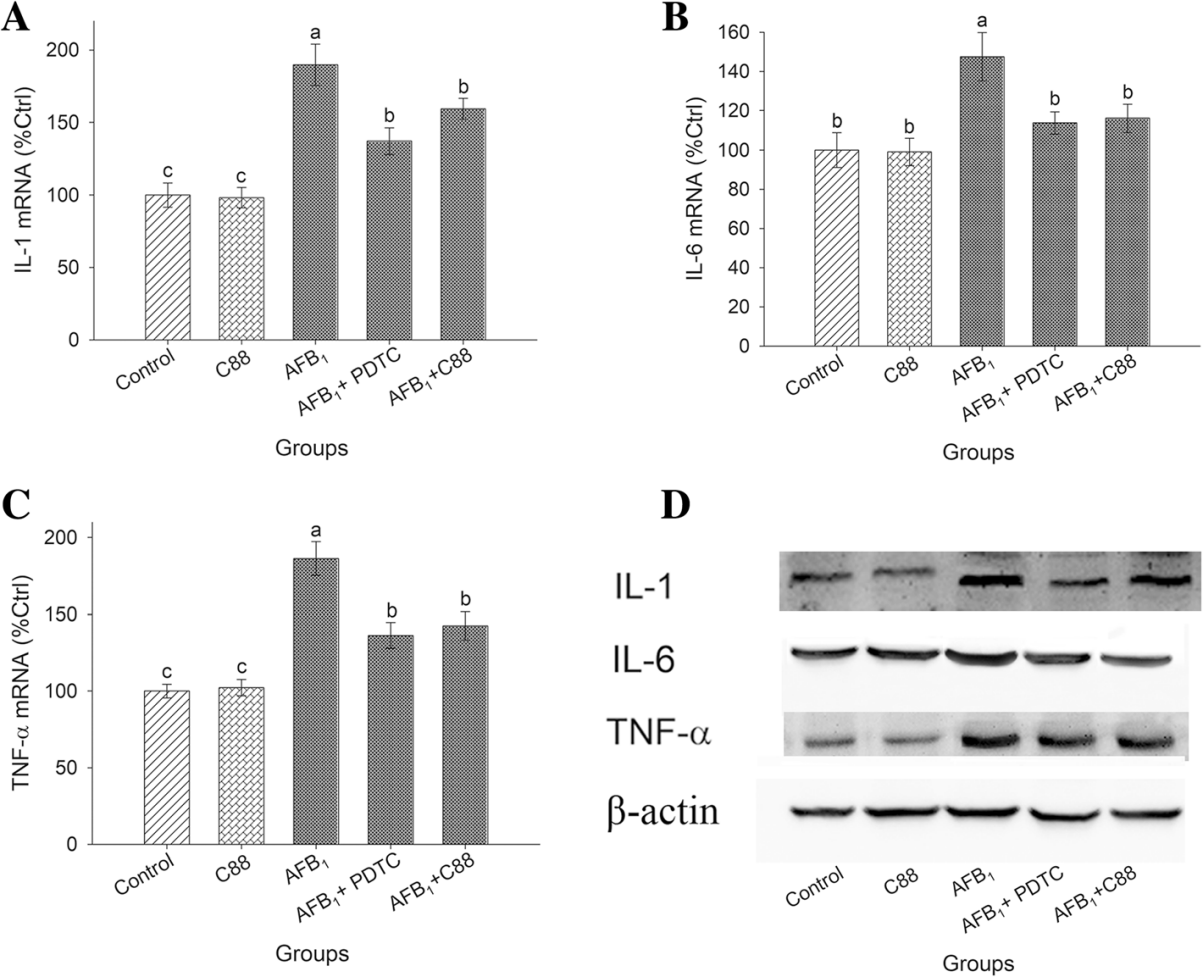
Reducing the production of pro-inflammatory cytokines through modulating the NF-κB signaling pathway. IL-1β (**a**), IL-6 (**b**) and TNF-α (**c**) gene expression was measured by RT-PCR. Expression of IL-1β, IL-6 and TNF-α (**d**) and β-actin was detected by Western blot analysis. β-actin was used as a housekeeping control. The results are expressed as mean ± S.D.; each data point is the average of 3 repeated measurements from 10 independently replicated experiments (*n* = 10). The different letters in the same rows mean significant difference (*P* < 0.05), the same letters in the same rows mean insignificant difference (*P* > 0.05)

The purpose of the neutralization trial was to investigate whether down-regulation of pro-inflammatory cytokines by *L. plantarum* C88 is specifically related to inhibition of NF-κB signaling pathways. Increased expressions of pro-inflammatory cytokines were observed in the AFB_1_ model group, while addition of *L. plantarum* C88 reversed this trend. Addition of Pyrrolidinedithiocarbamic acid (PDTC) led to decrease in the expression of IL-1β, IL-6, and TNF-α, indicating that NF-κB was strongly associated with the synthesis of pro-inflammatory cytokines (Fig. 3).

## Discussion

*L. plantarum* C88 exerts various probiotic properties, including ameliorating oxidative stress, improving intestinal barrier dysfunction, inhibiting intestinal endotoxin-mediated inflammation, blocking alcohol-induced chronic and acute liver damage. Hence, the underlying mechanisms of *L. plantarum* C88 on AFB_1_-induced chronic liver injury in mice were investigated in this study.

Cumulative evidence suggested that inflammatory response could facilitate structural and functional liver injury [19]. In fact, the increase of inflammatory cytokines was an important factor for causing liver cancer [20]. In this study, the levels of pro-inflammatory cytokines increased in the AFB_1_ model group, but the increase in the levels of inflammatory cytokines could be significantly prevented by *L. plantarum* C88. Our results are consistent with previous finding that *L. plantarum* supplementation could reduce TNF-α in the small intestines of AFB_1_-treated mice [21].

As a central regulator of cellular stress in hepatocytes, NF-κB regulates the processes of inflammation, apoptosis and cell injury [22]. Among the identified Toll-like receptors (TLRs), TLR2 and TLR4 are the major receptors for AFB_1_ recognition [23, 24]. The activation of TLR2 and TLR4 signaling led to degradation of IκB, NF-κB translocation to the nucleus, followed by NF-κB binding to the promoters of inflammatory genes and initiation of transcription in Kupffer cells [25]. In the present study, under AFB_1_ challenge, the mRNA expressions of TLR2, TLR4, and NF-κB were obviously up-regulated. However, the diet supplemented with *L. plantarum* C88 down-regulated the expression of NF-κB, reduced the level of NF-κB in the nucleus and up-regulated the expression of IκB in the liver. Another study indicated that *L. casei* weakened the lipopolysaccharide-induced overexpression of TLR4 and NF-κB mRNAs [26]. Ma et al. [27] proposed that AFB_1_-induced elevation of inflammatory cytokines was attributed to up-regulation of the NF-κB signaling pathway. Therefore, we hypothesized that *L. plantarum* C88 could diminish pro-inflammatory cytokines in the serum of mice, which might be related to the down-regulation of NF-κB expression. To confirm this hypothesis, neutralization test was performed. Pre-treatment with PDTC, an inhibitor of NF-κB, led to decline in the mRNA levels of IL-1β and TNF-α, indicating that the change of pro-inflammatory cytokine levels was positively correlated with the level of NF-κB, which was also verified by Western-blot. Similar results were obtained in the AFB_1_ + C88 group. Thus, *L. plantarum* C88 exhibited anti-inflammatory effects by down-regulating the NF-κB signaling pathway. Sun et al. [28] found that *L. paracasei* restrained the production of pro-inflammatory cytokines via inhibition of the TLR2/NF-κB signaling pathway. MiRNAs were shown to play a role in the regulation of the TLR2/NF-κB pathway in probiotic-mediated innate immune responses [29]. Probiotic genomic DNA was also found to have similar inhibitory functions [30]. Further studies are required to identify the exact structures of *L. plantarum* C88 that are responsible for inhibiting AFB_1_-induced inflammatory responses through NF-κB signaling pathways. Interestingly, the expression of TNF-α is regulated by the NF-κB signaling pathway, which, in turn, is activated by the combination of TNF-α and tumor necrosis factor receptor 1 (TNFR1) [22]. TNFR1, as the primary mediator of TNF-α, can be activated to trigger NF-κB and apoptosis signaling pathways [31]. Petrof et al. [32] showed that conditioned media from *L. plantarum* inhibited NF-κB activation from TNFR pathways. The present study focused on the mechanisms of TNF-α expression regulated by NF-κB in AFB_1_-induced mice, but the regulation of NF-κB by TNF-α is unclear. NF-κB regulates various apoptosis-related genes, including Bcl-2 families, TNFR-associated factors, JNK, etc. NF-κB could inhibit Caspase-8 expression and promote JNK activation by inducing the expression of IAP family, and thus play a role in anti-apoptosis [33]. In the early stage of viral hepatitis, NF-κB was activated, and subsequently induced hepatocyte apoptosis and acute liver failure by up-regulating the expression of Fas receptor [34]. Hence, the activation of the pro-apoptotic pathway or anti-apoptotic pathway by NF-κB was perhaps associated with differences in cell types and stimuli.

Apoptosis is related to inflammatory response. Previous researches have demonstrated that the mitochondrial pathways or the cell death receptor pathways were associated with excessive apoptosis of AFB_1_-induced hepatocytes. In this study, the proteins related to mitochondria-mediated apoptosis, such as Bax, Bcl-2 and caspase-3, were investigated. The pro-apoptotic protein Bax antagonizes the anti-apoptotic protein Bcl-2, which regulates the mitochondrial membrane permeability, and facilitates mitochondrial apoptotic factors, such as cytochrome C, into the cytoplasmic matrix to promote apoptosis. The protein kinase C (PKC), which is activated by hydrolysis of Caspase-3, executes the apoptotic process. In the present study, the levels of Bax and Caspase-3 were elevated, while the level of Bcl-2 was decreased in the liver of mice after oral administration of AFB_1_. Furthermore, the therapy groups reversed the above trend, indicating that *L. plantarum* C88 protected against excessive apoptosis induced by AFB_1_ through impeding the mitochondrial pathway. Zhang et al. [35] found that *L. rhamnosus* GG culture supernatant attenuated alcohol-induced hepatic apoptosis in mice. We also explored the cell death receptor pathways. Death receptors, such as Fas and TNF-α, bind to the associated death domains FADD and TRADD, respectively, and subsequently activate caspase-8 to induce apoptosis [36, 37]. Josse et al. [38] observed that the expression of Fas was increased after treatment of human hepatocytes with 0.05 μM AFB_1_. *L. paracasei* GMNL-32 protected cardiomyocytes from systemic lupus erythematosus mice via reducing the signaling pathway of Fas death receptor [39]. However, the changes in TRADD expression level in the liver after treatment with AFB_1_ have not been reported. In the present study, *L. plantarum* C88 restrained the increase in the mRNA expression levels of Fas, FADD, TRADD and Caspase-8 induced by AFB_1_, which demonstrated that *L. plantarum* C88 could protect liver tissue and restore liver cells from AFB_1_-induced hepatocyte apoptosis.

## Conclusions

The present study showed that *L. plantarum* C88 prevented AFB_1_-induced liver injury. *L. plantarum* C88 significantly decreased the levels of ALT and AST and ALP in serum, inhibited the secretion of pro-inflammatory cytokines, impeded the NF-κB nuclear translocations via enhancing the expression of I-κB, and ameliorated the excessive apoptosis in liver.

## Materials and methods

### Bacterial strain

*L. plantarum* C88 was cultured in Man–Rogosa–Sharpe (MRS) medium for 18 h at 37 °C. Thereafter, the bacterial density was adjusted to 10^10^ CFU/mL using phosphate-buffered saline (pH 7.2). Heat-killed *L. plantarum* C88 sample (10^10^ CFU/mL) was obtained by autoclaving the viable bacterial samples.

### Animals and experimental design

Male ICR mice, 6-week-old, body weight approximately 18–22 g, were supplied by by the Institutional Animal Care and Use Committee of Jilin University (SCXK 2015–0001). The mice were maintained in artificial illuminated (12 h light/dark cycles), constant temperature (25 °C) and environment humidity (50 ± 5%) room, with free access to food and water during the 1-week acclimation period. All mice received humane care. Ninety healthy ICR mice were randomly assigned to the six groups (*n* = 15, per group). The control mice received normal saline. The viable or heat-killed *L. plantarum* C88 group respectively received 4.0 × 10^10^ CFU/kg bw (body weight) viable or heat-killed *L. plantarum* C88 by gavage. The AFB_1_ group mice received 300 μg AFB_1_/kg bw, because of moderate toxicity symptoms was shown in the mice at this dose in the pre-experiment. In the therapy groups, the same dose of the viable or heat-killed *L. plantarum* C88 was administered after 300 μg AFB_1_/kg bw exposure (AFB_1_ + Viable or Heat-killed C88) for 21 days continuously. Twenty-four hours after the final treatments (i.e. day 21), the mice were anesthetized by inhalation of diethyl ether, blood samples were collected from retro-orbital venous plexus for the determination. After blood samples were collected, all mice were killed by cervical dislocation. The extracted blood samples were clotted and centrifuged (2000×g, 10 min, 4 °C) to obtain serum for subsequent analysis. The liver samples were divided into two parts. One half of the liver tissue was homogenized with cold physiological saline to prepare a 10% liver homogenate. The other half of the liver tissue was stored at − 80 °C for analysis of mRNA and protein expression levels (Additional file 4).

### Measurement of biochemical parameters in serum

The levels of ALT, AST, ALP, total cholesterol, triglycerides, total protein and albumin in serum were analyzed to evaluate liver damage. Alanine, aspartate and phenyl phosphate were used as substrates, respectively, to determinate the enzyme activities of ALT, AST and ALP in accordance with the manufacturer’s recommended procedure of assay kits (Nanjing Jiancheng, China). The absorbance was determined at 510.0 nm (ALT and AST) or 520.0 nm (ALP) using a microplate reader (Bio-Rad model 680, USA). The levels of total cholesterol, triglycerides, total protein and albumin in serum were measured as per the methods provided with the assay kits (Nanjing Jiancheng, China), using a microplate reader (Bio-Rad model 680, USA).

### Measurement of inflammatory cytokines in serum

IL-1β, IL-6, IL-8, IL-10, IFN-γ, and TNF-α levels in serum were determined by enzyme-linked immunosorbent assay (ELISA) as per the manufacturer’s recommended procedure of the ELISA kits (R&D Systems, USA).

### Measurement of Bax, Bcl-2 and Caspase-3 in liver

The ELISA kits (Cusabio, China) were used to detect the levels of Bax, Bcl-2, and Caspase-3 in liver. The absorbance was determined at 450.0 nm using a microplate reader (Bio-Rad model 680, USA).

### Analysis of DNA fragmentation in liver

The collected liver samples were homogenized in cold physiological saline. The DNA of liver tissue was extracted using the apoptosis DNA ladder detection kit (Dingguo Changsheng, China) as per the manufacturer’s instructions. The DNA fragmentation was assayed by electrophoresis in a 1.5% agarose gel and visualized by UV fluorescence after staining by ethidium bromide.

### Neutralization

As a potent inhibitor of NF-κB, pyrrolidine dithiocarbamate (PDTC) was used in the neutralization experiments. The isolation and culture of splenocytes were performed as previously described [18]. Splenocytes were divided into the control, C88, AFB_1_, AFB_1_ + PDTC and AFB_1_ + C88 groups. 0.1 μmol/ml PDTC (MedChemExpress, USA) and 10^10^ CFU/ml *L. plantarum* C88 was added to the AFB_1_ + PDTC and AFB_1_+ C88 groups, respectively, for 1 h. Then, normal saline or *L. plantarum* C88 were added to the control group and C88 group, respectively, while the other groups were exposed to 2 μg/ml AFB_1_ for 2d. The cells were collected and used to test the expression of IL-1β, IL-6 and TNF-α using real-time PCR analysis and Western blot analysis.

### Quantitative real-time PCR analysis

The procedure was consistent with a previous study [17]. Total RNAs were collected from frozen samples and reverse-transcribed into cDNA. Real-time polymerase chain reaction (RT-PCR) analysis was performed to determine the mRNA levels of specific genes on a LightCycler 96 Real-Time PCR system (Roche Diagnostics GmbH, Germany). The PCR conditions were 95.0 °C for 3.0 min, 40 cycles at 95.0 °C for 15.0 s, and 60.0 °C for 1.0 min. The primer sequences for RT-PCR are listed in Table 3. β-actin, a housekeeping gene, was used as an endogenous normalization control.

**Table 3:** Primer sequences for real-time PCR

Gene	Primer sequence	NCBI Reference Sequence:	References
NF-κB p65	Forward 5′ - GGACAGCACCACCTACGATG - 3′ Reverse 5′ - CTGGATCACTTCAATGGCCTC - 3’	NM_009045.4	Present study
I-κB	Forward 5’ - CAGGAGCCAAAACCGACAAC - 3′ Reverse 5′ - TGGTTGTCAGGTCTGCAATTTT - 3’	NM_001306222.1	Present study
Fas	Forward 5’ - CCAAACGGAAATTGCAGGGG - 3′ Reverse 5′ - AAGCACCAGTTCACAGATGGA - 3’	NM_001146708.1	Present study
FADD	Forward 5’ - TGCTCCACCTATCCACCAGA - 3′ Reverse 5′ - CAATGCGGAAGGCGATTGAG - 3’	NM_010175.6	Present study
TRADD	Forward 5’ - GAGCTGCTGGAGTGCAACTA - 3′ Reverse 5′ - GGTCCGGGTACTTAGAGGGT - 3’	NM_001033161.2	Present study
Caspase-8	Forward 5’ - CCAGACAGAGAAGGGGCTTG - 3′ Reverse 5′ - TCACTGCCCAGTTCTTCAGC - 3’	NM_001080126.1	Present study
IL-1β	Forward 5’ - TCGTGCTGTCGGACCCATAT - 3′ Reverse 5′ - GTCGTTGCTTGGTTCTCCTTGT - 3’	NM_008361.4	Present study
IL-6	Forward 5’ - GACAAAGCCAGAGTCCTTCAGA - 3′ Reverse 5′ - TGTGACTCCAGCTTATCTCTTGG - 3’	NM_001314054.1	Present study
TNF-α	Forward 5’ - GCGGAGTCCGGGCAGGTCTA - 3′ Reverse 5′ - GGGGGCTGGCTCTGTGAGGA - 3’	NM_001278601.1	Present study
β-actin	Forward 5’ - TGCTGTCCCTGTATGCCTCTG - 3′ Reverse 5′ - TTGATGTCACGCACGATTTCC - 3’	NM_007393.4	Present study

### Western blot analysis

The extraction and Western blotting analysis of total protein and nuclear protein from liver tissue were accomplished as previously described [17]. Protein was separated by gel electrophoresis and then transferred to nitrocellulose membranes (Merck Millipore, USA), which were probed using primary antibodies, including anti-TLR2, anti-TLR4, anti-NF-κB p65, anti-IκB, anti-Fas, anti-FADD, anti-TRADD, anti- Caspase-8 and anti-β-actin (Biosynthesis Biotech Co., China), for 12 h at 4 °C. Then the membranes were incubated with horseradish peroxidase-conjugated antibody for 1 h. ChemiScope 5600 image analyzer (Clinx Science Instruments, China) was used to quantify the antibody-bound proteins.

### Statistical analyses

Experimental results were numerically represented as means ± standard error. Data differences among the groups were analyzed using t-test. *P* < 0.05 was considered to be statistically significant difference.

## Additional files

Additional file 1: The raw datas of biochemical parameters in serum, include ALT, AST, ALP, total cholesterol, triglycerides, total protein and albumin. (XLSX 14 kb)

Additional file 2: The raw datas of IL-1β, IL-6, IL-8, IL-10, IFN-γ, and TNF-α in serum. (XLSX 15 kb)

Additional file 3: The raw datas of Bax, Bcl-2 and Caspase-3 in liver. (XLSX 13 kb)

Additional file 4: The ARRIVE Guidelines Checklist. (PDF 1067 kb)

## Data Availability

All data generated or analysed during this study are included in this published article and its supplementary information files.

## Abbreviations

AFB_1_: Aflatoxin B_1_
ALP: Alkaline phosphatase
ALT: Alanine aminotransferase
AST: Aspartate aminotransferase
FADD: FAS-associated death domain
Fas: Fatty acid synthetase receptor
HCC: Hepatocellular carcinoma
IARC: International Agency for Research on Cancer
IFN-γ: Interferon-γ
IL-1β: Interleukin-1β
IL-6: Interleukin-6
IL-8: Interleukin-8
IκB: Nuclear factor kappa B inhibitor
NF-κB: Nuclear factor kappa B
PDTC: Pyrrolidinedithiocarbamic acid
PKC: Protein kinase C
TLR2: Toll-like receptor 2
TLR4: Toll-like receptor
TLRs: Toll-like receptors
TNFR1: Tumor necrosis factor receptor 1
TNF-α: Tumor necrosis factor-α
TRADD: TNF receptor associated death domain
